# Current and future perspectives of mycotoxin research — report from the 42^nd^*Mycotoxin Workshop* (Online conference)

**DOI:** 10.1007/s12550-021-00439-7

**Published:** 2021-07-28

**Authors:** Svetlana Kalinina, Benedikt Cramer, Hans-Ulrich Humpf

**Affiliations:** Institute of Food Chemistry, Westfälische Wilhelms-Universität, Corrensstrasse 45, 48149 Münster , Germany

The 42nd *Mycotoxin-Workshop* 2021 was held on 31st May–2nd June, as an online conference. Unfortunately, last year’s *mycotoxin workshop*, which was planned in Brno (Czech Republic) had to be cancelled due to the COVID-19 pandemic. This year, an online forum for the presentation of new developments in mycotoxin research, as well as for the discussion of future perspectives in this field of research, was provided by the team of Prof. Dr. Hans-Ulrich Humpf, who is affiliated with the Institute of Food Chemistry, University of Münster. As always, the meeting was organised in cooperation with the *Society for Mycotoxin Research*. The scientific programme was scheduled for 3 days and contained nine main half-day sessions and six parallel short-talk poster sessions, covering contemporary mycotoxin research topics. This year, the *Mycotoxin Workshop* attracted the attention of almost 300 virtual participants from 31 countries worldwide, which resulted in 40 lectures and 69 short poster-style presentations. The online format of the *Mycotoxin Workshop* enlarged the geography of the members giving the opportunity to share the research with colleagues not only from EU, but also from Brazil, Canada, Chile, China, Egypt, Kenya, Saudi Arabia, South Africa, and the USA (Fig. 1).

The opening session was addressed by the president of the *Society for Mycotoxin Research*, Prof. Dr. Dr. habil. Manfred Gareis, and by Prof. Dr. Hans-Ulrich Humpf. Both speakers emphasized the importance of scientific exchange despite the COVID-19 pandemic. The opening ceremony was dedicated to the memory of Prof. Dr. Wentzel C. A. Gelderblom, one of the leading mycotoxin toxicologists significantly involved in the discovery of carcinogenic fumonisins, who suddenly passed away shortly before the *Mycotoxin Workshop*.

As in previous years, the main aim of the meeting was to combine various fields of research related to mycotoxins. Thus, the conference started with new developments in the field of human biomonitoring of mycotoxins highlighting the importance and prospect of novel methods for mycotoxin screening in physiological samples. The main focus was on exposure assessments of the population from various countries — Pakistan, Portugal, Chile, Germany, and France — to multiple mycotoxins, such as aflatoxins, deoxynivalenol, fumonisins, ochratoxin A, citrinin, and zearalenone. The increased interest of researchers to exposure scenarios through indoor environments was demonstrated during lectures covering the topics of mould infested building materials, and mycotoxin storage. Notably, gliotoxin was presented as a promising exclusive marker not only for the indoor microfungal dust contamination hazard but also for human *Aspergillus* infections. Although the analysis of mycotoxins by HPLC–MS/MS and HPLC-HRMS is becoming more and more established, many laboratories still face difficulties with significant matrix effects, sample purification and enrichment. An inspiring presentation on “in sample calibration” based on stable isotope labelled standards and the different probabilities for specific fragmentations completed the session “Analytical developments: Recent developments in the field of mycotoxin analysis”.

Toxicology, as a further prominent area in mycotoxin research, conversed during three sessions. Remarkably several lectures were about the application of cell culture and animal models to gain a deeper understanding of mycotoxin toxicity and modes of action on cellular and subcellular levels. *Fusarium* spp. and its metabolites became the most debated during the first session, while *Alternaria* toxins and ochratoxin A were in focus of the second session. Thus, for instance, ochratoxin A was revealed to induce pathological features of Parkinson’s disease. 17β-Hydroxysteroid dehydrogenase I was shown for the first time as a biological target of alternariol. Moreover, combined *Alternaria* toxins and their interference with pathways relevant for colonic immune responses, antioxidant capacity, and the structural integrity of intestinal cells were discussed. The final aspects of the toxicology sessions addressed the effects of mycotoxins on animals and plants, toxicity prediction, and strategies for detoxification. One upcoming topic was the distribution and absorption of mycotoxins by plants and possible consequences. Inhibitory effects of *Pseudomonas* as well as *Lactobacillus* on *Fusarium* and *Alternaria* growth were also discussed, highlighting the search for new effective biocontrol agents and strategies. The consequences of climate change for agriculture, namely elevated temperatures, increased CO_2_ concentrations, and drought stress, were in focus of a further session. Experimental results, as well as probabilistic models, indicate that a change of fungal colonization towards more drought-resistant *Aspergillus* fungi in the south of Europe has to be expected. Also, in Northern Europe climate change can lead to a shift of the traditional mycotoxin patterns and thus pose new risks and challenges to food and feed producers. Surely this topic will shift even more into scientific and public interest during the next years.

The secondary metabolism in filamentous fungi conversed in detail during the session “Genetics and metabolites: regulation of mycotoxin biosynthesis and fungal metabolites”. New insights into fusapyrone biosynthesis and self-protection mechanisms of the fumonisin producer *Fusarium* strains were discussed during the lectures. Novel data of *Penicillium expansum* exudates, so-called guttation droplets, analysis in terms of patulin production was also presented during this session. Phenylspirodrimanes, secondary metabolites from *Stachybotrys chartarum,* were shown as biologically active compounds.

By tradition, one of the most important goals of the *Mycotoxin Workshop* was to support young scientists. Therefore, almost two-thirds of the poster-style short lectures were presented by PhD students. Furthermore, this year for the first time, postdoctoral researchers and junior group leaders had the opportunity to chair the sessions (Fig. 1).

**Fig. 1:**
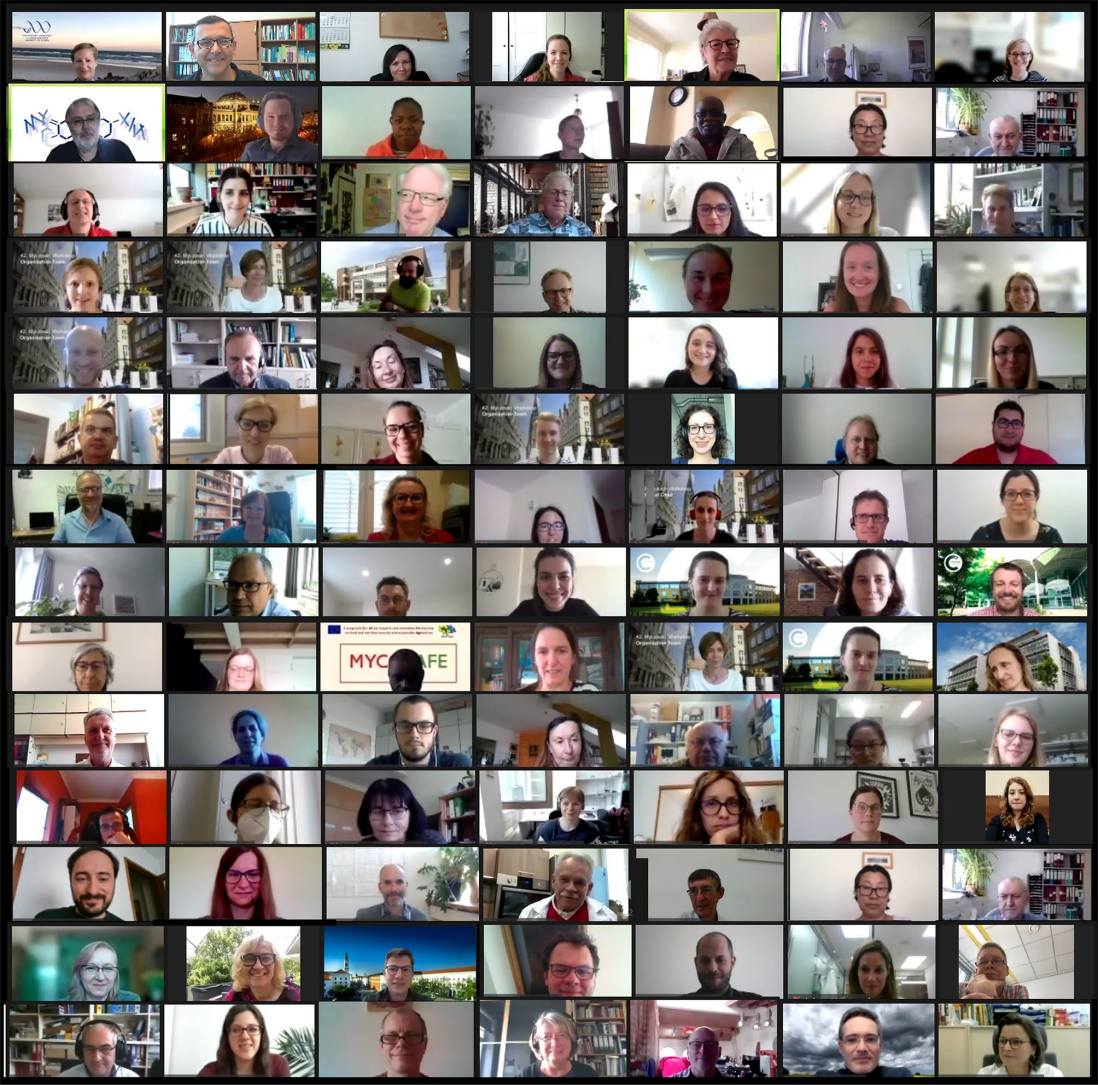
Participants with “camera on” during the 42nd *Mycotoxin Workshop*

## Brigitte Gedek Science Award 2020/2021

A highlight of the *Mycotoxin Workshop* was the awarding of the Brigitte Gedek Science Prize. Due to the COVID-19 crisis the ceremony could not take place last year and had to be postponed to 2021.

The award is donated by the Brigitte and Wolfram Gedek Foundation and is intended to promote scientific work in the field of mycotoxinology.

**Fig. 2:**
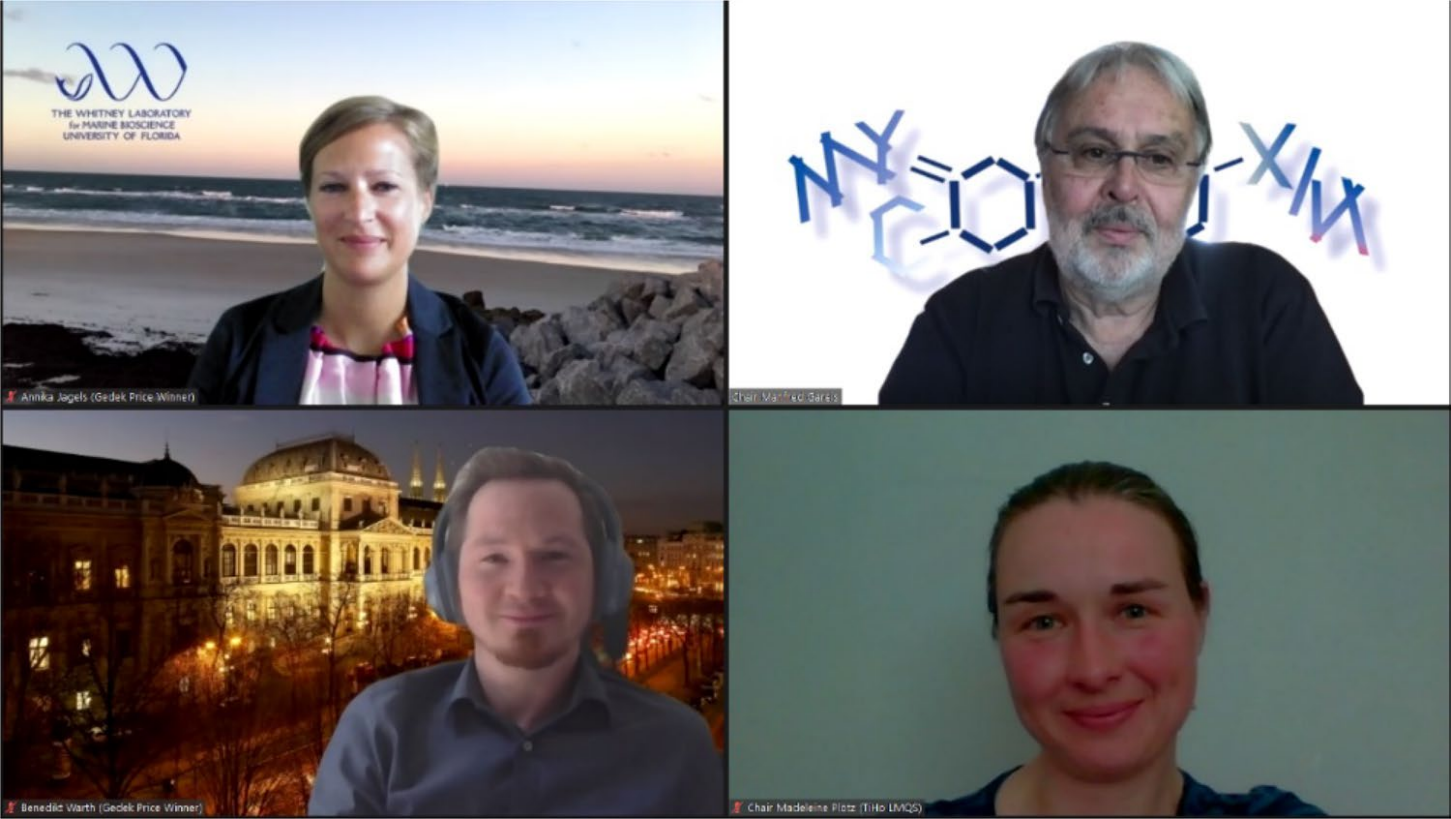
The winners of the Brigitte Gedek Research Award 2021 Dr. Annika Jagels (top left) and Prof. Dr. Benedikt Ward (bottom left) during the presentation of the award by the President of the *Society for Mycotoxin Research*, Prof. Dr. Dr. habil. Manfred Gareis (top right) and Vice-President Prof. Dr. Madeleine Plötz (bottom right)

This year’s Science Prize was split and awarded equally to two laureates: Dr. Annika Jagels from the University of Münster, Germany, and Prof. Dr. Benedikt Warth from the University of Vienna, Austria (Fig. 2).

The award committee honoured the doctoral thesis of Ms. Jagels “Isolation and structure elucidation of new secondary metabolites from *Stachybotrys* species and human exposure studies” and the publication of Mr. Warth et al. on the “Transfer and Metabolism of the Xenoestrogen Zearalenone in Human Perfused Placenta” (Environ. Health Persp., 2019).

This highly prestigious prize in the field of mycotoxin research is endowed with 10,000 Euro and given biennially by the *Society for Mycotoxin Research*. The award is open to scientists worldwide, but the application of the candidate must be supported by a member of the *Society for Mycotoxin Research*. All details for the next call will be provided on the home page of the *Society for Mycotoxin Research* (www.mycotoxin.de).

## General meeting of the *Society for Mycotoxin Research*

The annual meeting of the *Society* was held during the 42nd *Mycotoxin Workshop*. The board of directors reported about the number of members and the financial situation of the *Society*. Currently, the *Society* has about 180 members from 26 countries with increasing tendency especially from young scientists. The *Society for Mycotoxin Research* is a non-profit organisation focusing on scientific excellence and exchange in the field of mycotoxin research and supporting particularly young scientist. Members benefit from a free print and online subscription of the official journal of the *Society*, *Mycotoxin Research*, as well as a highly reduced registration fee for the *Mycotoxin Workshop*. Prof. Dr. Ewald Usleber, the Editor in Chief of *Mycotoxin Research,* gave a short report about the current situation with the official journal of the Society. *Mycotoxin Research* and its impact for the scientific community continue to develop very well. For example, the impact factor (Clarivate Analytics) increased from 3.1 in 2019 to 3.8 in 2020.

For his excellent job as Editor in Chief of *Mycotoxin Research*, Prof. Dr. Ewald Usleber was elected as honorary member of the *Society*.

According to the bylaws of the *Society*, the Board of Directors was newly elected. The current President of the *Society*, Prof. Dr. Dr. habil. Manfred Gareis, who served the *Society* for 20 years since its foundation in 1997, wished to step back from his position. Prof. Dr. Hans-Ulrich Humpf expressed his willingness to follow Manfred Gareis as President. In a unanimous vote, the following Board of Directors was (re)-elected for the period 2021–2023:

President: Prof. Dr. Hans-Ulrich Humpf, Münster, Germany.

Vice President: Prof. Dr. Madeleine Plötz, Hannover, Germany.

Secretary: Dr. Karsten Meyer, Freising, Germany.

Treasurer: Dr. Benedikt Cramer, Münster, Germany.

Prof. Humpf thanked Prof. Gareis for his outstanding and long-lasting support of the *Society for Mycotoxin Research*.

## Announcement: 43rd *Mycotoxin Workshop* 2022 in Toulouse, France

The 43rd *Mycotoxin Workshop* will take place on May 30–June 1, 2022, in Toulouse, France. The conference will be organized by the groups of Dr. Isabelle Oswald, Prof. Florence Mathieu and Dr. Mohamed Haddad in cooperation with the *Society for Mycotoxin Research*. For further information concerning registration, abstract submission, deadlines, and fees, please check the website of the *Society for Mycotoxin Research* (www.mycotoxin.de).

